# Biosecurity at Cattle Farms: Strengths, Weaknesses, Opportunities and Threats

**DOI:** 10.3390/pathogens10101315

**Published:** 2021-10-13

**Authors:** Véronique Renault, Marie-France Humblet, Phuong N. Pham, Claude Saegerman

**Affiliations:** 1 Research Unit in Epidemiology and Risk Analysis Applied to Veterinary Sciences (UREAR-ULiege), Fundamental and Applied Research for Animal Health (FARAH) Centre, Faculty of Veterinary Medicine, University of Liege, B-4000 Liège, Belgium; vrenault@uliege.be; 2 Department of Occupational Safety and Health, Biosafety and Biosecurity Unit, University of Liege, B-4000 Liège, Belgium; mfhumblet@uliege.be; 3 Department of Emergency Medicine, Brigham and Women’s Hospital, Harvard Medical School, Harvard Humanitarian Initiative, Harvard T.H. Chan School of Public Health, Boston, MA 02115, USA; ppham@hsph.harvard.edu

**Keywords:** biosecurity, cattle, Belgium, context, SWOT analysis, conceptual framework

## Abstract

Biosecurity is a key component of any animal and public health strategy and disease prevention and control programs. This study reviewed the main findings of different studies implemented from 2015 to 2021 to analyse the biosecurity situation at Belgian cattle farms, including attitudes and behaviours of cattle farmers and rural veterinarians regarding biosecurity measures. Specifically, the objective was to perform a SWOT (strengths, weaknesses, opportunities and threats) analysis of the situation and propose a new conceptual framework improving the level of biosecurity in the cattle sector. Biosecurity in cattle farming remains relatively low and faces multiple challenges. Its future improvement requires the different stakeholders to agree on shared goals and objectives and to carefully consider animal, public and environmental health, as well as socioeconomic and cultural factors. Further cost efficiency studies are required to identify the most important biosecurity measures and convince the stakeholders of their utility and benefits. Cattle farmers rely mainly on rural veterinarians for technical guidance and consider them as trustful informants. To be more effective in promoting these good practices, rural veterinarians need a proper guidance from the authorities, a proper training on biosecurity and communication, as well as an enabling environment.

## 1. Introduction

“Prevention is better than cure.” This quote attributed to Desiderius Erasmus in around 1500 CE certainly applies to both human and animal health. Over the years, infectious diseases have caused a huge impact on both animal and public health. Due to the progress in science and epidemiology, many measures to prevent and control the spread of these diseases have been identified and promoted. Biosecurity has been defined by the World Health Organization and the Food and Agriculture Organization as “a strategic and integrated approach to analysing and managing relevant risks to human, animal and plant life and health and associated risks for the environment” [1]. It is a key element of the European Union Animal Health Strategy [2]. As part of the One Health approach, strengthening biosecurity in the different animal production systems is also important to preserve the public and environmental health.

**Figure 1:**
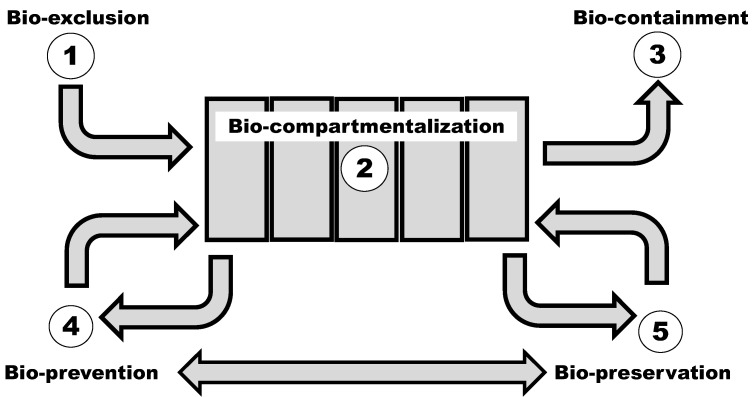
Biosecurity principles and compartments in animal facilities (Adapted form: Saegerman, Dal Pozzo and Humblet, 2012).

Biosecurity in animal production systems includes the measures that can be implemented by the animal producers at the farm level in order to manage the risks of infectious diseases in their premises. It represents the basis of disease control measures against endemic and exotic diseases. Biosecurity in animal productions systems can be divided into five stages or compartments in order to highlight its importance not only in terms of animal health, but also its role in protecting public health and the environment (Figure 1) [3]. These five compartments are: (i) bio-exclusion, biosecurity measures (BSM) preventing the introduction of a pathogen at a farm, (ii) bio-compartmentalization, BSM preventing the spread of a pathogen within the farm, (iii) bio-containment, BSM preventing the spread of the pathogen to other farms or premises, (iv) bio-prevention, BSM preventing the spread of zoonotic pathogens to humans, and (v) bio-preservation, BSM preventing environmental contamination. Each BSM can be related to one or several biosecurity compartments. For example, quarantining newly purchased animals contributes to bio-exclusion while a proper carcass disposal system contributes to bio-compartmentalization, bio-containment, bio-prevention and bio-preservation. 

Based on the international and national animal health authorities, some BSM contributing to a disease eradication or control program are mandatory (e.g., winter screening in Belgium to detect potential shedders or carriers and tests purchased for some diseases) while others should be implemented on a voluntary basis. 

Proper implementation of biosecurity in the animal production system has been strengthened over the years, especially in more intensive production systems such as pig and poultry industries. Nevertheless, based on several studies (Table 1), the level of implementation of BSM at cattle farms remains particularly low despite the intensive communication and awareness-raising campaigns performed on the matter over the last years. 

These findings highlight the need to investigate further the reasons of low implementation of BSM by cattle farmers and the farmers’ attitudes and perceptions towards biosecurity (BS). Empirical evidence shows that targeted interventions are more effective when theoretical models take into account the different determinants of behaviour and behaviour changes [11]. Such studies are necessary to properly understand the farmers’ mindset, identify the specific factors which will convince them to start the change process and be able to properly motivate farmers to change their behaviour [12]. The health belief model (HBM) is the most frequently used in studies related to health and farmers’ behaviours [12]. It has been considered as “a useful framework for understanding individual differences in health behaviour patterns and for designing behaviour change intervention” since the 1970s [13]. Based on the HBM, the intention to perform a behaviour is related to five determinants: the risk perception in terms of probability and severity, the perception of the behaviour benefits or outcomes, the perception of barriers to the behaviour implementation or to the outcome achievement and the health motivation which was included later in the model [14]. Each of the five perceptions of the HBM are assumed to be influenced by different demographic and psychological variables. Several health communication messages targeting the HBM variables to change behaviours were proven successful over the years [15] and a consensus statement was published in 1977 to endorse the HBM framework as a model to better understand the sociopsychological determinants of health and health-related behaviours [16].

The objective of this article was to review the existing studies on biosecurity in Belgian cattle farming in order to analyse the strengths, weaknesses, opportunities and threats (SWOT analysis) prevailing in the actual Belgian context in order to propose a new conceptual framework of actions and conditions necessary to ensure a wider adoption of BSM by cattle farmers, increase the farmers’ resilience towards infectious diseases and answer the major public health challenges regarding zoonoses and antimicrobial resistance.

## 2. Existing Studies on Biosecurity in Cattle Farming

Several studies were implemented from 2014 to 2021 in order to better understand the situation of biosecurity at Belgian cattle farms and the actual prevailing context (Table 2). 

### 2.1. Main Outcomes of the Existing Studies on Biosecurity in Cattle Farming

In the current globalized market, Belgian local producers are in competition with foreign producers whose production costs are lower along with less attention to quality assurance and control regulations. Furthermore, local Belgian agricultural products in most supermarkets have been priced higher than imported products. Belgian producers often have to sell their products cheaper than at fair market prices. For example, in 2017, the price of Belgian locally produced milk was €0.29, while the production costs were estimated at €0.46 per litre [26]. The beef market is not favourable either to European cattle farmers with a reported marginal profit and a decreasing ratio between the meat value and the input costs [27]. This little-to-no profit margin and the repeated agricultural crisis need to be considered when developing production regulations and policies that ensure high quality of animal food and food products to answer the consumers’ demand and public health challenges. 

A farm survey [21] found that Belgian cattle farmers recognize the importance and benefit of preventive measures in comparison to curative medicine. Most of the farmers interviewed considered their biosecurity level as satisfactory. Nevertheless, the overall implementation level of BSM at farms remains low. The farmers expressed several constraints. The measures’ feasibility (26%), a relevance perceived as low in the actual context (19%) or an efficacy perceived as poor (19%) were identified as the main reasons for not implementing a specific BSM [21]. The perceived benefits of BSM were also identified among the main factors determining their implementation [24]. As documented by several researchers [4,28], the variations and contradictory biosecurity recommendations by different regulation bodies coupled with the lack of easily accessible publications of such regulations makes it confusing and difficult for farmers to comply with them. The Belgian farming context does not make an exception as several farmers mentioned having received contradictory information from agents from the same authority. A lack of consensus on the importance and/or priority of BSM to be implemented also prevails among rural veterinarians. Another example of the impact of confusing and contradictory information is also illustrated by a 2014 study which highlighted several contradictions in the epidemiological surveillance system in Belgium [18]. Indeed, an exhaustive list of infectious animal and zoonotic diseases is lacking, and there are differences between the list of diseases effectively monitored and the diseases which should be monitored, based on advice of the scientific committee of the Belgian Federal Agency for the Safety of the Food Chain (FASFC) or the documented cost–benefit analysis. The actual level of information awareness of animal health professionals in Belgium is not sufficient to provide a uniformed and proper level of information to cattle farmers. For example, unlike for intensive pig and poultry farming, there is no official website or overall legislation mentioning an exhaustive list of mandatory biosecurity measures that farmers should implement at their cattle farms. Mandatory measures are only mentioned in some disease-specific laws and ministerial or royal decrees such as the royal decree related to foot and mouth disease [29]. The same issues have been raised by an analysis performed on the EU legislative framework on animal health which highlighted the following [30]:

- The lack of clear links with other legislation such as public health, food safety and environmental protection and lack of consistency between the different animal health legislation pieces;
- The lack of overall strategy and the need to focus on increased biosecurity;
- The lack of a horizontal law with the obligations being spelled out in different legal acts.

As mentioned in previous studies [4,28] and confirmed in the Belgian context [22,24], it is necessary to clarify the priority diseases to be targeted by surveillance and control programs. It is also essential to identify the priority BSM recommended for animal health professionals in order to harmonise and provide uniform communication to cattle farmers. Ideally, such clarification and harmonisation should be performed through a participatory process including animal health experts, including private veterinarians, as well as cattle farmers to ensure the acceptability of recommendations.

The new European Union animal health law provides a legal framework to biosecurity actions and measures [31]. It emphasizes that “biosecurity is a key prevention tool” and clarifies that “the biosecurity measures adopted should be sufficiently flexible, suit the type of production and the species or categories of animals involved and take account of the local circumstances and technical developments”. Nevertheless, this text does not provide any specific recommendation in terms of biosecurity measures to be prioritized and/or be made mandatory. It defines biosecurity as “the sum of management and physical measures designed to reduce the risk of the introduction, development and spread of diseases to, from and within animal population or an establishment, zone, compartment, means of transport or any other facilities, premises or location” which does not reflect the importance of biosecurity in terms of public and environmental health and might lead to the omission of these important aspects in any future document or policy. The importance of animal welfare and of a safe and stress-free environment is also often omitted while it is an important component of animal health and represents a growing concern for the consumers.

### 2.2. Priority Diseases and Biosecurity Measures for the Cattle Farming Sector

Several studies mention that the reasons for the low adoption of the recommended BSM might come from the difference of perspective and objectives prevailing between the authorities, veterinarians and cattle farmers [5,21,24,32]. These differences often lead to a perception of insufficient or inadequate public policies on biosecurity [33]. Due to the difference of objectives in communication messages, cattle farmers might not be interested in communication and will therefore not process the information or seek additional guidance. Some major differences between the priority diseases listed by the World Organisation for Animal Health (OIE), the priority zoonosis listed by the European Centre for Disease Prevention and Control (ECDC) and/or the priority diseases listed by the Belgian authorities and some of the most frequent diseases encountered at cattle farms (online veterinary survey) were indeed identified in Belgium [20]. The latter are more likely to be the farmers’ priority but were not listed in any of the previous prioritization studies (e.g., multifactorial diseases such as mastitis, interdigital dermatitis and diarrhoea). When considering the six priority diseases (i.e., bovine respiratory diseases (BRD), bovine respiratory syncytial virus (BRSV), bovine viral diarrhoea (BVD), infectious bovine rhinotracheitis (IBR), Q fever and salmonellosis) for the Belgian authorities and cattle farmers [20], it appears that their transmission pathways (based on the diseases typology) illustrate all possible pathways. Their related BSM therefore include all BSM. Therefore, trying to emphasize the BSM related to these six diseases will not permit the prioritisation of BSMs. This negative finding will hopefully encourage the sanitary authorities to improve efforts aiming at preventing the potential introduction and spread of other notifiable diseases important to the national or international animal and public health authorities but not considered as a priority by cattle farmers. Indeed, the adoption of new practices by cattle farmers could be increased if the sensitization messages were to focus on the BSM aimed at controlling the diseases considered as important by cattle farmers. Therefore, despite the different disease control objectives, animal health authorities and cattle farmers could reach an agreement on the determination of priority BSM. A table designed thanks to the literature review shows which disease(s) among a selection of 47 diseases (Appendix A Table A1) are addressed by each BSM (Appendix A Table A2). It is an operational tool which can facilitate easy identification of BSM in order to timely and effectively control and prevent targeted diseases.

The benefits or outcomes of BSM are the most important elements to consider in any effort to motivate farmers’ uptake of recommendations [24]. According to 10 out of the 14 studies reviewed, these factors are associated with BSM implementation. Although labelled differently, “benefits” (e.g., perceived importance, attitude towards BSM or positive outcomes of behaviour) should be considered more as a ratio rather than an absolute number. The perceived “benefits” of a BSM will be high when its perceived positive outcomes are considered higher than the perceived constraints or related costs. The positive outcomes could be related to herd health and productivity as well as to public health, environmental health or animal welfare. Indeed, the positive perception of cattle farming by the general public and their health responsibility appears to be of increasing importance for cattle farmers, as shown in previous studies [24,27,34,35,36].

The benefits could also be measured in terms of cattle farmers’ resilience to infectious diseases. Proper knowledge of the actual level of implementation and the possible improvements to be adopted in case of outbreaks could help mitigate different disease outbreaks and increase the capacity of a farmer to mitigate the impact of disease outbreaks in the country or the surroundings either by preventing the contamination of their herd or by better containing the disease. Studying the effect of different BSM implementation of the farmer’s resilience towards infectious diseases could effectively convince them to adapt their behaviour based on risk levels. 

As mentioned by some cattle holders during the field interviews, “no BSM is too costly as long as it is useful and effective. It depends of the positive economic impact of the measure.” This highlights the need for evidence-based and cost effectiveness studies, as mentioned also in previous studies [37,38,39], and the recognised lack of knowledge to identify the worthwhile BSM to recommend [7]. Most studies recommending biosecurity practices do not provide strong evidence of their efficacy or cost effectiveness. Besides, the existing studies on disease cost efficiency usually target a single practice or focus on the prevention of specific diseases [4]. A pilot study performed on a small sample of farms tested a methodology to estimate the overall benefits of a higher biosecurity level [40]. It showed interesting results as a higher biosecurity level was significantly correlated with a BVD-free status, a lower mortality rate in adult cattle (over 24 months) and young calves (0–7 days). With strong evidence, reproduced on a larger scale with additional steps to convert cattle heads preserved or gained (through higher reproductive parameters) at farms with high biosecurity levels could bring the needed evidence and support the communication by providing clear cost effectiveness evidence. The data collection of an eventual longitudinal study of cattle farms with different biosecurity levels could also be used in order to predict the eventual economic losses due to lower implementation of biosecurity by using a stochastic model predicting the herd evolution overtime based on different mortality rates. Such methodology was previously used successfully to analyse the cost–benefit ratio of the semestrial contagious caprine pleuropneumonia vaccination campaigns in Kenya [41]. 

### 2.3. Guidance and Technical Advice to Cattle Farmers

With reported multiple and sometimes conflicting recommendations, it is necessary to improve the technical guidance offered to cattle farmers in terms of biosecurity. In the absence of evidence-based cost effectiveness analyses, farmers might be tempted to adopt the easy to implement BSM which may not always be the most effective or relevant in their case [28]. There is a need for standardized and improved communication among animal health authorities and the different providers of animal health services to prevent any further confusion of farmers and harmonise the message [32]. The cattle farmers’ level of trust towards national authorities and control instances is described as low in many studies and thus needs to be improved [42,43] for proper adherence of farmers to disease control programs and legislation. It also appears from several studies that recommendations should be provided on a case-by-case basis, considering the specific farm environment and context in order to better address the farmers’ priorities. 

The role of veterinarians in providing such advice is essential as they are considered as the main reference for biosecurity as well as a trustful source on information [38,43,44,45]. Nevertheless, as mentioned in the introduction, it requires a shift from rural veterinarians, which is not easy for several reasons including the unproven efficacy of BSM, the absence of common understanding and agreement on the key recommendations and inadequate biosecurity public policies [33]. A study targeting Belgian rural veterinarians showed that most veterinarians do consider biosecurity as a priority (88%) but less than 50% have attended continuous training on biosecurity and/or got informed on the topic in the course of personal studies [22]. In addition, among the things to improve, the study highlighted the need for technical advice on biosecurity. For example, the recommendation regarding animal purchase is often restricted to the mandatory tests and does not include other important diseases in the Belgian context such as Mortellaro disease or mastitis.

Resources on the relationship between the different BSM and infectious diseases can be useful to rural veterinarians (Appendix A Table A1 and Table A2). It is also the case for Chapter 14 of the book on biosecurity for animal producers and veterinary medicine “Transmission of Cattle Diseases and Biosecurity in Cattle Farms” [46]. Nevertheless, these tools are not sufficient. As part of the BOBIOSEC project funded by the Belgian Federal Public Service Health, Food Chain Safety and Environment, an online risk-based scoring system to quantify biosecurity in cattle production was jointly developed by the University of Liège and the University of Ghent (available at https://biocheck.ugent.be/en) (accessed on 6 October 2021) [47]. More specifically, the Biocheck Cattle relies on a questionnaire that was developed based on the outcomes of face-to face questionnaires used in previous studies [21,23]. These studies helped identify not only the BSM to be included in the scoring systems, but also the measures to be excluded in order to shorten the questionnaire (e.g., questions without a clear score, questions correlated within the same category or questions with less than 15% variation in the application and not stressed by multiple sources in the literature review). All the BSM categories and subcategories were weighted based on the experts’ opinion in order to elaborate a risk-based scoring system accessible to all farmers and veterinarians free of charge. After completing the survey, the user obtains an overall biosecurity score and scores per category; that score can be compared to the average score of her/his country. Paid additional functions are provided by the system, too, such as personalized and automatically generated feedback, continuous monitoring of the farm BS level with downloadable reports, comparison and sharing as well as online training. The basic Biocheck application, free of charge, allows the user to identify farm weaknesses and the main areas of improvements. It also provides a benchmarking system that permits the comparison of their situation to their peers. Such aspects could promote the adoption of new BSM by cattle farmers and help veterinarians to perform a standardized and repeatable risk assessment with personalized recommendations. Nevertheless, the system still has some inherent limitations. The weighting system remains subjective as it is based on the experts’ opinion, but it also provides the general weight, while the weights usually depend on the targeted diseases. That could be a bias for farmers who have a disease-specific objective. The list of the BSM used in the questionnaire was reduced but is still quite extensive as it includes between 69 and 214 questions depending on the type of farm (veal, beef or dairy). This takes time to complete and can deter its use, especially if assessments need to be repeated over time by famers. 

Other tools in relation to biosecurity were developed in different countries; they might be of interest in Belgium if we consider the findings of previous studies that highlighted the farmers’ interest for specific and technical guidance to achieve personal animal health objectives [21,48]. Veterinary herd health management is becoming increasingly important but needs to be based on the farmers’ main goal and a cooperative strategy defined commonly with the farmer to ensure the implementation of recommendations and a long-term success [49]. This strategy should be based on a few mandatory measures which can be controlled and farm-specific recommendations based on the farmers’ risks and needs. In Australia, for example, a smartphone application was developed to help farmers to develop their own biosecurity plans (https://www.farmbiosecurity.com.au/toolkit/farmbiosecurity-app/) (accessed on 6 October 2021): they can select different actions from a list of suggested BSM and monitor their progress. Such tools are interesting and should be looked into in more detail to better promote biosecurity at cattle farms. 

### 2.4. Other Biosecurity Stakeholders Who Should Be Considered and Sensitized

Professional visitors (e.g., veterinarians and salesmen) represent another group of key stakeholders in mitigating biosecurity risks, as highlighted by several studies. Some findings highlighted the fact that farmers are aware of this risk but do not act on it. This is mainly due to the fact that they trust the professionalism of the visitor (mainly for veterinarians and artificial inseminators) or do not feel in the position to impose on them restrictive measures as they do need their services (for cattle salesmen) [21]. In the actual Belgian context, it is clear that the risk of introduction of infectious diseases to a farm by professional visitors is high as cattle farmers perceive it as the responsibility of the visitor, or as something they do not have any control over. If rural veterinarians consider they properly manage the risk of disease introduction to the farms they visit, there is still a large room for improvement as they tend to overestimate their implementation of bio-exclusion and bio-containment measures [22]. It appears from different studies implemented in Belgium that the misunderstanding between veterinarians and cattle farmers goes beyond the animal health objectives discussed above, but also lies in their shared responsibility to ensure correct bio-exclusion [21,22]. When considering, for example, the poor implementation of hygienic measures upon entering farms (e.g., cleaning boots and changing clothes), most veterinarians mention the absence of cleaning facilities at farms as well as the farmers’ responsibility to provide farm-specific clothing while farmers consider it is the veterinarian’s professional responsibility. There is a need for a better communication between veterinarians and farmers to clearly understand the respective expectations and agree on a common way forward as solutions are applied by some farmers (farm-dedicated clothing) or veterinarians (own mobile disinfection unit). Cattle farmers should also be empowered and feel in position to impose restrictive measures on visitors without any negative impact.

The attitude and beliefs of other professional visitors should be studied as well in order to better identify the risks and possible mitigation measures to be established. Among them, cattle salesmen represent a major risk as, according to the farmers’ survey, most of them do not take specific hygiene measures and, as it is the case for veterinarians, cattle farmers are reluctant to condition their access to the stables as they depend on them. A survey aimed at determining the level of awareness regarding biosecurity and BSM among other professionals working with farmers would strengthen the biosecurity levels at cattle farms.

## 3. Discussion

**Figure 2:**
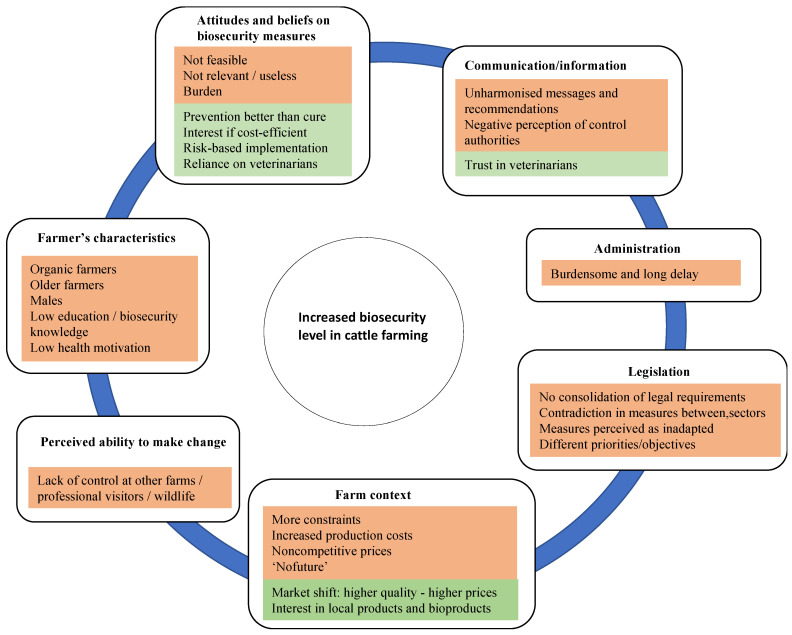
Proposed conceptual model explaining the factors affecting the implementation of biosecurity measures at Belgian cattle farms. In orange, negative-effect factors; in green, positive-effect factors.

Several factors negatively affect the implementation of BSM at cattle farms (Figure 2), according to several studies conducted in Belgium. These factors are linked either to the farmer’s characteristics, attitudes and beliefs or to the farm context, the administrative and legal context. Based on this analysis, several recommendations can be made to the different actors in order to improve the biosecurity level at cattle farms.

In terms of research, there is an urgent need for evidence-based cost efficiency studies in order to identify the priority BSM and convince the farmers of their cost efficiency. As mentioned previously, the actual knowledge does not allow the identification of worthwhile BSM and leads to general confusion as different actors recommend different measures [28]. It appears from previous studies that even the veterinarians considered as the main informants on technical guidance have mentioned the lack of information on BSM efficiency. This finding was confirmed through the exchanges with cattle farmers during a farm survey [21] as many of them reported receiving contradictory information from different entities (e.g., FASFC agents versus veterinarians). In addition, the absence of a common goal and objective among the national authorities, veterinarians and cattle farmers adds to the confusion and the cattle farmers’ negative perception of the national control and eradication programs, often considered as irrelevant [18,28,32,49]. Based on the transtheoretical model of behaviour change, any information considered as irrelevant or not answering a specific need of the recipient is not processed and used. In order for any communication to be effective in changing behaviour, it should raise the interest of the target group by answering their needs.

To ensure the adequacy of the recommended national control and eradication programs and mandatory BSM, a proper analysis should be performed by a group comprising experts from different sectors, including cattle farmers, to jointly identify the priorities to be addressed and the related BSM. It is indeed essential to agree on common objectives in terms of animal, public and environmental health. If not, the measures will be perceived as irrelevant or not important by cattle farmers or might end up in contradiction with the recommended measures from other sectors. The identified measures should be relevant to the needs of cattle farmers, acceptable, feasible and shared with other sectors in order to avoid possible contradictions (e.g., the need to remove bushes and vegetation for vector control while environmental rules promote natural hedges and, in some areas, forbid vegetation clearing). As for the One Health approach, BSM should be considered in a holistic approach and, as suggested by a recent study, as “a unified concept to integrate human, animal, plant and environmental health” [50]. Negative impact of some preventive treatments on the environment or human health has been documented in the past. Some examples include the development of (multi)drug resistance linked to the preventive use of antibiotics in some intensive farming system [51], the contamination of the environment related to treatments of animals with acaricides [52] and the negative effects on beneficial insects consecutive to the use of chemical larvicides in the control of vector breeding sites [53]. Such negative impacts could be avoided in the future, showing the importance of having the One Health approach and build interconnections between health, agriculture and the environmental sector and considering the natural and social sciences which can facilitate the adoption of BSM by the population [50]. These aspects are clearly taken into consideration by the European Green Deal which policy areas include, among other things, biodiversity (measures to protect the ecosystem) and food safety under the terminology “From Farm to Fork”.

The identification of common goals and objectives through a participatory and intersectoral approach should also help farmers to regain some trust towards national control authorities. It is also essential to define the roles and responsibilities of the different stakeholders in order to improve the farmers’ perception of their health responsibility and ability to make change.

Effective training and communication to farmers should be implemented, ideally by trustful sources such as veterinary practitioners or farmers’ associations, in order to promote biosecurity and the major BSM. Such communications should focus on the factors determining the implementation of BSM, which were identified as follows: BSM benefits and cost effectiveness, as well as responsibility of cattle farmers towards animal, public and environmental health. Special attention should be brought to organic farmers as their perception of BSM benefits and health motivation are lower, while these two constructs are the key factors determining the implementation of BSM. The development of online and mobile applications for farmers to perform self-evaluations and get personalised feedback on biosecurity are also of major interest and should be promoted as the actual tool, Biocheck Cattle, is promising but has several constraints and limitations. As demonstrated by previous studies [22,24], the shift from curative to preventive medicine implies repositioning of the rural veterinarian and a much-needed different approach. While more consultants and private counsellors provide paid herd management and biosecurity advice, veterinarians still perceive that farmers are reluctant to pay for such services. Furthermore, even if these services are free of charge, the veterinarians’ investment in time and resources for such advice should be considered. There is an urgent need to change the rural veterinarians’ and cattle farmers’ perceptions on that issue in order to pursue the shift from curative to preventive medicine. Indeed, most biosecurity pieces of advice provided by the veterinarians are still an answer to animal health problems reported at farms and/or are restricted to mandatory measures [22].

There are two more challenges that have an important impact on the farmers’ behaviour regarding BSM: (i) unfavourable farming context and (ii) farmers’ lack of control on the selected bio-exclusion measure. The actual farming context is not favourable for cattle farmers as imported animal products and subproducts coming from countries with lower production costs and constraints compete with local products. The farmers need to identify coping strategies to increase their competitiveness on the market and their profits. The shift in the consumers’ profile, with an increasing demand in local products and an apparent willingness to purchase quality products at higher prices, is encouraging and might change the negative opinion on the future of cattle farming. National and international initiatives supporting local product consumption and promoting a fair price to the producers should be encouraged. The last challenge is the apparent farmers’ lack of control over some bio-exclusion measures as no measure seems to be totally efficient in preventing interactions of cattle with wildlife and cattle farmers perceive as difficult the control of professional visitors. Regular monitoring and surveillance programs should be implemented to mitigate the risk of disease introduction through wildlife as the environmental and demographic changes will certainly increase contacts between domestic animals and wildlife in the future. Previous studies [22,24] showed that rural veterinarians are well aware of the risk they represent and have a professional responsibility to prevent any disease transmission. Nevertheless, the basic BSM are not always complied with and their shared responsibility is not always recognised. The situation is most likely similar or even worse with other professional visitors such as cattle salesmen or feed suppliers. Further studies should be implemented to clearly identify the risk related to each professional visitor as well as different workshops and training in order to make them aware of biosecurity issues and the risk they represent. Such workshops and training should be the responsibility of the national authorities or farmers’ associations.

## 4. Conclusions

As illustrated by the current COVID-19 pandemic likely caused by zoonotic transmission [54], the interest in biosecurity has increased over the years, and its concept is becoming more important due to the multiple threats and increased risk related to the demographic changes, environmental changes, globalization and increased international exchange and travel. The biosecurity level has been strengthened in the Belgian intensive production systems (e.g., pig and poultry industry), with clear mandatory measures and recommendations integrated in a common legislative document, while the biosecurity level at cattle farms remains low and faces multiple challenges. Its improvement will require the different stakeholders to take actions as recommended above and a clear legal framework providing the list of obligations and recommendations in terms of biosecurity at cattle farms. Further studies obtaining strong evidence demonstrating BSM cost efficiency and identifying the priority BSM to recommend can convince the stakeholders of their utility and benefits. The measures highlighted in this article should also be implemented in order to serve as a basis for the decision-making process of different actors.

## Figures and Tables

**Table 1:** Main findings of different studies related to the implementation level of biosecurity measures in cattle farms.

Reference	Country	Herd Type	Type of Questionnaire and the Main Findings
Brennan and Christley, 2012 [4]	North West England	All	Face-to-face interviews (*n* = 56) Many BSM “implemented infrequently or not at all” Implementation mainly influenced by cost, unproven efficacy, knowledge of BSM
Nöremark, Frössling and Lewerin, 2010 [5]	Sweden	All	Postal questionnaire (*n* = 518, with 192 cattle farms) “Many had biosecurity routines that were not satisfactory from an infectious disease prevention perspective”, “Only 10% of cattle farmers reported that they isolated animals before introduction into the herd” and “Most farmers perceived their on-farm biosecurity as ‘sufficient’ (72%)”Lower implementation at cattle farms and in smaller herds; low perception of the disease risk and insufficient knowledge of disease transmission pathways and BSM
Sayers et al., 2013 [6]	Republic of Ireland	Dairy	Tele- and hardcopy surveys (*n* = 444) Implementation rate higher in large herds, among older farmers and in regions with higher cattle density
Sanderson, Dargatz and Garry, 2000 [7]	USA	Beef cow/calf producers	Personal interviews (*n* = 1190) “Producers do not always adjust management practices such as vaccination schedules and quarantine procedures appropriately to minimize this risk” “At this point, we lack adequate data to know which, if any, biosecurity practices are worthwhile”
Sahlström et al., 2014 [8]	Finland	All	Mailed questionnaires + online survey (*n* = 1236) Implementation at cattle farms is lower than at pig farms; the farmers were satisfied with their on-farm biosecurity even though the biosecurity level was not particularly high Better implementation in larger herds and at dairy farms
Hoe and Ruegg, 2006 [9]	USA, Wisconsin	Dairy	Mailed questionnaires (*n* = 587) Overall, most management practices were associated with the herd size, but many beliefs regarding important dairy farm issues were consistent Better implementation in larger herds
Brandt et al., 2008 [10]	USA, Central Plains	Feed yards	Interviews with feed yard personnel (*n* = 106) Overall, low implementation level of biosecurity measures Low implementation related to the lack of knowledge on risks and biosecurity measures and to a low perception of the cost effectiveness of biosecurity measures Better implementation in larger herds

Legend: BSM, biosecurity measures.

**Table 2:** Non-exhaustive list of studies performed in Belgium to analyse different aspects of biosecurity in the cattle sector.

Title	Reference
A survey on biosecurity and management practices in selected Belgian cattle farms	[17]
Evaluation de la surveillance épidémiologique Belge en santé animale	[18]
Determinants of risk behaviour: effects of perceived risks and risk attitude on farmer’s adoption of risk management strategies	[19]
Classification of adult cattle infectious diseases: A first step towards prioritization of biosecurity measures	[20]
Biosecurity practices in Belgian cattle farming: Level of implementation, constraints and weaknesses	[21]
Rural veterinarian’s perception and practices in terms of biosecurity across three European countries	[22]
Biosecurity practices in Belgian veal calf farming: Level of implementation, attitudes, strengths, weaknesses and constraints	[23]
Cattle farmers’ perception of biosecurity measures and the main predictors of behaviour change: The first European-wide pilot study	[24]
Exploring cattle movements in Belgium	[25]

## Data Availability

No new data were created or analyzed in this study. Data sharing is not applicable to this article.

## Appendix A

**Table A1:** List of the 47 diseases selected.

Code	Disease		
1	Acute and subclinical mammitis	25	Paratuberculosis
2	Anaplasmosis/ehrlichiosis	26	Q fever/Coxiellosis
3	Anthrax	27	Schmallenberg disease
4	Aujeszky’s disease	28	Secondary infections
5	Babesiosis (bovine)	29	Tuberculosis (bovine)
6	Bluetongue	30	Botulism
7	Bovine herpesvirus 4	31	Bovine respiratory diseases
8	Bovine spongiform encephalopathy	32	Bovine viral diarrhoea (BVD)
9	Brucellosis	33	Coccidiosis
10	Campylobacteriosis	34	Cryptosporidiosis
11	Crimean–Congo hemorrhagic fever	35	Diarrhea/enteritis
12	Cryptococcosis	36	*Escherichia Coli* verotoxic
13	Cysticercosis	37	Enterotoxemia (*Clostridium* spp)
14	Dermatophytosis/mycosis	38	Giardiasis
15	Distomatosis	39	Infectious bovine rhinotracheitis (IBR)
16	Echinococcosis	40	Infectious bovine keratoconjunctivitis
17	Enzootic bovine leucosis	41	Intestinal parasites
18	Foot and mouth disease	42	Lice and ectoparasites
19	Interdigital and digital dermatitis	43	Listeriosis
20	Leptospirosis	44	Necrobacillosis (laryngitis)
21	Lyme disease/borreliosis	45	Rabies
22	Metritis (non-specific pathogen)	46	Salmonellosis (nontyphoidal)
23	Neosporosis	47	Scabies
24	Papillomatosis		

**Table A2:** Biosecurity measures efficient to prevent the introduction or spread of a selection of 47 diseases (listed in Appendix A Table A1).

DISEASE CODE (See Appendix A Table A1)	1	2	3	4	5	6	7	8	9	10	11	12	13	14	15	16	17	18	19	20	21	22	23	24	25	26	27	28	29	30	31	32	33	34	35	36	37	38	39	40	41	42	43	44	45	46	47	Total
BIOSECURITY MEASURES																																																
**1. Related to animal movements**																																																
Closed herd/no movements	1			1		1	1		1						1		1	1	1	1			1		1	1			1		1	1	1	1	1	1		1	1	1		1			1	1	1	**27**
Not taking part in cattle exhibitions	1			1		1	1		1									1							1	1			1		1	1	1	1	1	1		1	1	1								**18**
All-in/all-out system of each age group and each separate stable	1			1		1	1		1									1	1						1	1			1		1	1	1	1	1	1		1	1	1		1				1	1	**22**
Ensuring free source or origin/no importation of infected animals	1	1		1		1	1	1	1		1				1		1	1	1	1			1		1	1			1		1	1		1	1	1		1	1	1		1			1	1	1	**29**
Premovement testing (against specific diseases)	1	1		1		1	1		1						1		1	1	1	1			1		1	1			1		1	1		1	1			1	1	1		1			1	1	1	**26**
Quarantine (3 weeks, separate area or building (3 m distance)) and testing for entering or reentering animals	1	1	1	1	1	1	1		1		1						1	1		1			1		1	1			1		1	1		1	1	1		1	1	1	1	1			1	1	1	**29**
Testing for entering or reentering animals	1	1		1		1	1		1		1						1	1					1		1	1			1		1	1		1	1			1	1	1		1			1	1	1	**24**
Separate area or building (3 m distances) for quarantine	1		1	1		1	1		1								1	1		1			1		1	1			1		1	1	1	1	1	1		1	1	1		1			1	1	1	**26**
Reducing commingling when purchasing	1		1	1		1	1		1									1	1	1					1	1			1		1	1	1		1		1		1							1		**19**
Divide calves in high- and low-risk groups based on the veal calve risk classification				1		1											1		1						1				1		1	1	1						1							1		**11**
Good transport conditions: safely, in a clean truck with a decent loading ramp, no overcrowding, calm handling, as short as possible, not passing through a sorting center	1			1		1			1					1				1	1										1		1	1	1	1	1		1	1	1			1	1			1	1	**20**
**2. Related to vertical or venereal transmissions**																																																
No breeding animals shared with other farms						1	1		1	1							1			1					1	1			1			1							1									**11**
Check semen status before insemination						1			1								1								1																							**4**
Artificial insemination						1			1	1							1			1					1							1							1									**8**
**3. Prophylactic measures**																																																
Vaccination						1												1								1				1	1	1			1	1	1		1	1					1			**12**
Deworming strategies													1		1																										1							**3**
Preventive treatments		1			1															1						1					1			1	1			1		1		1						**10**
Regular hoof trimming by professionals (twice/year)																			1																													**1**
Regular foot bathing																			1																													**1**
**4. Vector control: prevent introduction of contaminated vectors/environment contamination**																																																
Tick control		1			1						1						1				1					1																						**6**
Mosquito/biting fly control		1				1											1							1			1	1												1		1					1	**9**
Identification of contaminated soils/pastures to prevent their usage			1										1																																			**2**
Rodent control program																				1						1					1			1				1					1			1		**7**
Destroy snail habitats/prevent access to snail habitats															1																																	**1**
**5. Prevent direct contact with eventual external shedders/carriers**																																																
Prevent contact at pastures with animals of neighbouring farms and wildlife (pigs and ruminants) (simple or doubles fences)		1		1	1		1		1									1		1					1				1		1	1	1	1	1	1		1	1	1	1	1	1		1	1	1	**24**
Closed housing/locked doors (prevent contact with pets, carnivores, rodents,… in stables)									1							1				1			1		1				1		1	1	1	1	1	1		1	1		1		1		1	1		**18**
Proper carcass disposal, avoid exposure to scavengers			1						1							1		1					1							1										1								**7**
Prevent dispersion of biological fluids during sample collection			1			1			1		1						1	1																														**6**
**6. Prevent contamination of food and water from external shedders/carriers**																																																
Storage of food in clean and closed structures to prevent their contamination				1				1	1			1	1			1				1			1		1					1	1					1							1			1		**14**
Clean water and feed troughs regularly							1		1				1			1				1					1				1	1	1	1	1	1	1	1		1	1		1		1			1		**19**
No access to surface water/prevent access to running or stagnant water at pastures													1		1	1				1			1		1					1			1													1		**9**
Equipment for the handling of manure should not be used for feed																				1					1						1	1	1	1	1	1		1	1		1					1		**12**
Cleaning and disinfection of feeding utensils									1				1			1				1			1		1				1		1	1	1	1	1	1		1	1		1		1			1		**18**
Identification and proper disposal of contaminated feed			1	1				1				1	1			1									1					1													1					**9**
**7. Prevent contamination by visitors**																																																
Access restriction for visitors + visitor control and registration	1			1					1								1	1								1			1		1	1	1	1	1	1		1	1			1	1			1	1	**19**
In-house or clean boots and clothes for visitors (availed by the farmer)	1			1					1									1						1		1		1	1		1	1	1	1	1	1		1	1		1	1	1			1	1	**21**
Personal working hygiene of professional visitors (hand hygiene, visitor’s own boots/clothes, etc.)	1						1		1				1					1						1		1		1	1		1	1	1	1	1	1		1	1		1	1	1			1	1	**22**
Usage of a disinfection footbath	1						1		1									1						1		1		1	1		1	1	1	1	1	1		1	1		1	1	1			1	1	**21**
Deny access to stables to cattle salesmen	1						1		1									1								1			1																			**6**
Vehicle access restriction/no vehicles in areas where animals are kept/pass by, separate access routes	1			1					1									1								1			1		1	1	1	1		1		1	1		1		1			1		**16**
Footbaths and hand washing facilities between compartments	1						1		1				1					1						1		1		1	1		1	1	1	1	1	1		1	1		1		1			1		**20**
**8. General management**																																																
Monitoring and recording.	1	1	1	1	1	1		1	1	1			1		1	1	1	1	1	1		1	1	1	1	1	1		1		1	1	1	1	1	1		1	1				1			1		**33**
Keep an up-to-date animal identification and recordkeeping register with animal health data	1	1	1	1	1	1		1	1	1			1		1	1	1	1	1	1		1	1	1	1	1	1		1		1	1	1	1	1	1		1	1							1		**32**
Constant surveillance and monitoring	1	1	1	1	1	1		1	1	1			1		1	1	1	1	1	1		1	1	1	1	1	1	1	1		1	1	1	1	1	1		1	1				1			1		**34**
Systematic control 5–6 weeks after parturition at high-risk farms (for metritis)																						1		1																								**2**
Identification and elimination/segregation of carriers/infected animals by regular testing	1	1			1	1	1	1	1	1							1	1		1			1	1	1	1			1		1	1		1	1			1	1	1						1		**24**
Maintain resistant breeds or endemic stability		1			1										1									1							1			1	1		1	1					1	1			1	**12**
Work organisation	1								1					1				1						1					1																			**6**
Working from young to old animals	1													1										1	1						1	1	1	1	1	1		1	1							1		**13**
Individual daily calf checks														1										1							1		1	1	1			1								1		**8**
Avoid excessive stress or stressful events	1													1										1				1	1		1		1	1	1		1	1	1				1	1		1		**15**
No equipment or vehicles shared with other farms									1									1						1		1			1			1							1		1					1		**9**
Bedding and flooring	1								1	1									1					1	1	1					1	1	1	1	1	1		1	1							1		**16**
Bedding/litter removal; keeping fresh and clean beddings	1						1		1	1				1					1					1	1	1		1			1	1	1	1	1	1		1	1		1		1			1		**21**
No recycling of bedding	1								1	1									1					1	1	1		1			1	1	1	1	1	1		1	1		1					1		**18**
Cemented floors/concrete flooring									1	1									1					1	1			1			1		1	1	1	1		1			1		1					**14**
Presence of rubber mats on the floor																			1					1				1															1					**4**
Proper disposal of manure from other farms within 500 meters																								1	1					1		1		1		1		1			1					1		**9**
Avoid piling manure																								1	1						1					1												**4**
Housing	1								1	1				1	1		1	1	1			1		1	1	1		1	1						1	1			1									**17**
Housing density	1								1	1				1	1		1	1	1			1		1	1	1		1	1		1	1	1	1	1	1	1	1	1	1	1	1				1	1	**28**
Good ventilation and air quality (positive pressure ventilation of > 15 cubic ft. per minute per calf	1													1					1					1				1	1		1	1	1	1	1		1	1	1					1				**15**
Maintaining a dry environment where possible	1													1					1					1	1	1		1	1																	1		**9**
Tie stall or stanchion facilities														1										1		1		1			1				1											1		**7**
House the animals per sex, no mixed groups																								1				1			1						1		1									**5**
Proper feeding	1													1								1		1				1	1	1	1	1	1		1		1				1		1	1				**15**
Well-balanced ration	1						1							1								1		1				1	1		1	1	1		1		1				1		1	1		1		**16**
Good feeding procedures	1						1							1										1				1	1	1	1	1	1		1		1				1		1	1		1		**16**
Control of adequate feed intake	1						1							1								1		1				1	1		1	1	1		1		1				1		1	1		1		**16**
Grazing practices													1		1					1				1	1	1			1												1							**8**
Integrated grazing management: shifting the animals every 7–14 days, no regrazing before 60 days, dose and move system															1									1																	1							**3**
Extensive grazing (beef)																								1					1												1							**3**
Zero grazing (dairy)													1											1													1		1		1		1					**6**
Pasture drainage															1									1																	1							**3**
Avoid sharing or renting pastures																				1				1	1	1													1		1							**6**
Mowing																								1																	1							**2**
Ploughing under manure before animals go to a pasture																								1						1											1							**3**
Biological control of helminths													1			1								1																	1							**4**
**9. General hygiene practices**																																																
Cleaning/disinfection of all possibly contaminated equipment	1			1		1	1		1	1			1	1		1	1	1	1	1		1	1	1	1	1		1	1		1	1	1	1	1	1		1	1		1		1			1		**31**
Cleaning stables before introduction of new calves, steam or hot water, thorough drying on multiple days	1						1		1	1				1				1	1	1					1	1		1	1		1	1	1	1	1	1		1	1		1	1	1			1	1	**25**
Sanitary vacancy ("vide sanitaire")	1						1			1				1				1	1	1					1	1		1			1	1	1	1	1	1		1	1		1	1	1			1	1	**23**
Cleaning and disinfection of equipment after each usage (calving, milking,…)	1					1	1		1	1				1			1	1	1	1		1		1	1	1		1	1		1	1	1	1	1	1		1	1		1		1			1		**27**
Regular hand cleaning and disinfection ( especially between age groups)	1						1		1	1				1				1	1	1		1		1	1	1		1	1		1	1	1	1	1	1		1	1		1		1			1		**25**
Proper cleaning and disinfection of surgical instruments and needles between animals	1	1			1	1	1		1	1	1			1			1	1	1	1		1		1	1	1		1	1			1							1									**21**
Animal transport vehicle and other vehicles leak-proof and cleaned and disinfected before entry through separate access routes				1					1									1		1					1	1			1		1	1	1	1	1	1		1	1		1	1	1			1	1	**20**
**10. Management of sick or quarantined animals**																																																
Only allow healthy animals on common pastures (testing)		1			1													1							1							1									1	1					1	**8**
Quick recognition, isolation and treatment of sick animals	1	1			1		1		1	1				1	1		1	1	1	1		1	1	1	1	1	1		1		1	1	1	1	1	1		1	1		1		1			1		**30**
Sick animals treated last	1						1		1					1					1					1	1	1			1		1	1	1	1	1	1		1	1				1			1		**19**
Quarantine facilities and work organisation	1								1					1			1	1						1	1	1			1		1	1	1	1	1	1		1	1				1			1		**19**
Separate quarantine stable-building, capacity at least 2% of the farm	1								1					1			1	1						1	1	1			1		1	1	1	1	1	1		1	1				1			1		**19**
Separate boots and impermeable clothing for the quarantine stable	1								1					1			1	1						1	1	1			1		1	1	1	1	1	1		1	1				1			1		**19**
A washing installation for the quarantine stable	1								1					1				1						1	1	1			1		1	1	1	1	1	1		1	1				1					**17**
Changing gloves for each sick animal	1								1					1				1						1	1	1			1		1	1	1	1	1	1		1	1				1					**17**
Daily observation									1															1	1	1			1		1	1	1	1	1	1		1	1				1					**14**
Separate housing of relapses and chronic cases									1																1	1			1		1	1																**6**
Injectable medication instead of an oral one (to control the actual uptake and dosage)																										1					1																	**2**
Effective and applicable treatment protocols and evaluation of the protocols																										1				1	1		1		1				1	1			1	1				**9**
Hospital ration with water and hay ad libitum, high level of protein and energy, vitamins and minerals																										1					1		1		1				1				1					**6**
Frequent and thorough cleaning of quarantine and hospital pens and their feed and water places																										1			1		1	1	1	1	1	1		1	1		1		1					**12**
**11. Parturition**																																																
Testing all cases of abortion									1											1			1			1						1																**5**
Maternity pen	1								1	1									1			1				1		1			1	1		1	1	1		1	1							1		**15**
Maternity pen separated from other animals	1								1	1									1			1				1		1			1	1		1	1	1		1	1							1		**15**
Existence of the maternity area with a sufficient amount of individual calving pens	1								1	1									1			1				1		1			1	1		1	1	1		1	1							1		**15**
Maternity pen designed for easy cleaning and drainage	1								1	1									1			1				1		1			1	1		1	1	1		1	1							1		**15**
Not using maternity pens for sick animals	1								1	1												1				1		1			1	1		1	1	1		1	1							1		**14**
Always someone present at the moment of calving	1								1	1												1						1			1				1	1												**8**
Cleaning and disinfection	1								1	1										1		1		1		1		1			1	1			1	1			1							1		**14**
Cleaning of the calving/abortion area (maternity pen/stables/box) before and after each calving/abortion	1								1	1										1		1		1		1		1			1	1	1		1	1			1							1		**15**
Cleaning and disinfection of the udder and the vulva	1								1	1										1		1		1				1			1	1	1		1	1			1							1		**14**
Cleaning and disinfection of hands before and after abortion and/or calving	1								1	1										1		1		1		1		1			1	1	1		1	1			1							1		**15**
Cleaning and disinfection of the obstetric material before and after abortion and/or calving	1								1	1										1		1		1		1		1			1	1	1		1	1			1							1		**15**
Immediate calf care	1																											1			1				1													**4**
Immediate clearing of airways																												1			1				1													**3**
Immediate separation of the calf from the mother (keep the calf with the cow for 24 hours (oldest))	1																							1							1			1	1	1		1	1							1		**9**
Navel dipping in a clean vessel with fresh disinfectant																												1			1				1				1									**4**
Immediate and proper disposal of fetal membranes and tissues after abortion and/or calving							1		1											1			1			1		1				1																**7**
**12. Calf management**																																																
Calf feeding									1								1								1						1	1	1		1	1								1				**9**
Good quality and quantity colostrum within rgw first 6 hours	1					1			1								1								1			1	1		1	1	1	1	1	1	1	1						1		1		**17**
Proper supply of milk (quantity and quality)	1																1														1	1	1	1	1		1	1						1				**10**
Avoid feeding of infected milk/pasteurization of fed milk	1								1								1								1			1	1	1	1	1		1	1		1	1					1	1				**15**
Temperature control of the milk given																														1	1				1		1											**4**
Gradual supply of concentrates and hay to adapt to a new diet																															1			1	1		1							1		1		**6**
Hutches/calf pen																	1								1			1			1	1		1	1	1		1						1				**10**
Hutches should be placed in an outdoor environment, situated to minimize weather impact																												1			1	1		1	1	1		1						1		1		**9**
Hutches should be placed 1.25 m apart																									1			1			1	1		1	1	1		1						1		1		**10**
Hutches should be cleaned, preferably steamed, disinfected and thoroughly dried before housing new calves (including underneath)																									1			1			1	1	1	1	1	1		1						1		1		**11**
Daily cleaning of bedding and housing of calves (stress-free, dust-free)																												1			1	1		1	1	1	1	1	1				1	1		1		**12**
Specific equipment																									1						1	1		1	1			1						1				**7**
Use of one bucket per calf with a teat																																1	1	1	1			1						1		1		**7**
Cleaning the buckets after each feeding																									1							1		1	1			1						1		1		**7**
Use of an oesophageal feeder only when necessary																															1																	**1**
Calf groups management	1																1								1			1	1		1	1	1	1	1	1	1	1								1		**14**
Calves and young stock separated from older animals and other age groups																	1								1			1			1	1	1	1	1	1		1	1							1		**12**
Temperature and humidity control <15 °C in the calf stable																	1								1			1			1	1	1	1	1	1	1	1			1			1		1		**14**
**13. Dairy management**																																																
Equipment	1								1																	1																						**3**
Regular control and maintenance	1																																															**1**
Immediate replacement of broken or cracked milk tubes	1																																															**1**
Wash and sanitize equipment after each milking	1								1															1																								**3**
Automatic milking system	1																									1																						**2**
Milking operations	1								1															1																								**3**
Teats clean and dry	1																							1																								**2**
Eventual teats disinfection before milking (dipping)	1																							1																								**2**
Examine foremilk	1																							1																								**2**
Teat disinfection after teat cups removal (dipping)	1								1															1																								**3**
Healthy young cow first, then older cows and infected cows last	1								1															1																								**3**
Ensure cows remain standing after milking ( fresh feed and water)	1																																															**1**
Establish goals for udder health and monitor their achievement	1																																															**1**
If purchaseing a lactating cow: isolation, separate/last milking and bacteriological culture	1																																															**1**
Separate first calf heifers from multiparous animals	1																																															**1**
Good and balanced nutrition	1																																															**1**
Clip flanks and udder	1																																															**1**
Preventive treatments	1																																															**1**
Monitoring of SCC	1																																															**1**
Dry period <4 days	1																																															**1**
Appropriate management of clinical mastitis	1																																															**1**
Culling of cows with chronic/nonresponsive intramammary infections	1																																															**1**
**14. Animal workers from the farm**																																																
Prevent contact with a farmer or a worker with cloven hoofed animals from other farms									1									1							1	1																				1		**5**
Personal working hygiene of a worker/farmer (boots, clothes, hands,…)	1								1		1							1						1	1	1			1		1	1	1	1	1	1		1	1		1	1	1			1	1	**21**
Regular training of animal keepers	1	1	1	1	1	1		1	1	1	1		1	1	1	1	1	1	1	1	1	1	1	1	1	1	1		1	1	1	1	1		1		1	1	1				1	1	1	1		**38**
Animal per person ratio as low as possible	1																	1											1		1	1	1		1				1							1		**9**
**15. Prevent human contamination (zoonosis)**																																																
Raw milk/milk products only from certified farms	1								1																	1			1																			**4**
Meat inspection/properly cook meat before consumption													1			1																																**2**
**16. Prevent environmental contamination**																																																
Manure treatments or spreading in the absence of wind only																										1																						**1**
**Number of measures adressing the disease**	**91**	**17**	**11**	**23**	**13**	**26**	**30**	**8**	**79**	**31**	**8**	**2**	**18**	**29**	**17**	**14**	**36**	**47**	**34**	**39**	**2**	**26**	**20**	**69**	**68**	**73**	**6**	**52**	**63**	**14**	**100**	**89**	**67**	**74**	**94**	**69**	**22**	**74**	**76**	**16**	**42**	**21**	**47**	**23**	**10**	**79**	**21**	
